# Warning to Ladies

**Published:** 1874-11

**Authors:** 


Interesting to Ladies who Wear False Hair.—A young girl, remarkably cleanly and neat in her personal appearance, came to the editor lately in great trouble and alarm. For some days she had suffered the most intolerable itching of the head, neck and body, and after consulting a physician and getting a prescription for some medicine "for the blood," she discovered that her head was swarming with lice. On inquiry as to her employment, the source of the vermin was discovered. She was employed in a hair factory.—*Pacific Med. and Surg. Journal.*

